# Deciphering the association between the Life’s Essential 8 and infertility: insights into depression, inflammation, and metabolic mediation

**DOI:** 10.3389/fendo.2025.1451030

**Published:** 2025-02-21

**Authors:** Shichao Cui, Li Li, Weibing Qin, Wensheng Liu, Xingming Zhong

**Affiliations:** NHC Key Laboratory of Male Reproduction and Genetics, Guangdong Provincial Reproductive Science Institute (Guangdong Provincial Fertility Hospital), Guangzhou, China

**Keywords:** cardiovascular health, infertility, Life’s Essential 8, inflammation, depression, metabolism

## Abstract

**Background:**

The Life’s Essential 8 (LE8) score has been associated with various health outcomes, but its relationship with female infertility remains unclear.

**Methods:**

This study investigated the relationship between LE8 and infertility in women aged 20-45 years using the National Health and Nutrition Examination Survey (NHANES) data from 2013 to 2020. Weighted multifactorial logistic regression models were utilized to examine the association between the LE8 factors and their two subgroups [health behavior score (HBS) and health factor score (HFS)], as well as depressive status and infertility. Nonlinear relationships were examined using weighted restricted cubic spline (RCS) regression. Subgroup analysis and mediation analysis of depression, metabolic, and inflammation further elucidated the relationships. Sensitivity analysis was conducted to ensure the robustness of the findings.

**Results:**

A total of 2,182 participants were included in this study, with 304 in the infertility cohort. Multifactorial regression analysis revealed significant negative correlations between LE8 and its subgroup HFS with infertility. Among the eight subscales, scores for sleep health, body mass index, and blood glucose were significantly negatively correlated with depression, while Patient Health Questionnaire-9 (PHQ-9) scores showed a positive correlation with infertility. Weighted RCS regression modeling indicated no nonlinear relationships between LE8, depression, HFS, HBS, and infertility. Mediation analyses suggested that depression scores, systemic immune inflammation index (SII), and uric acid (UA) mediated the association between LE8 and infertility.

**Conclusion:**

Higher LE8 scores, indicating better cardiovascular health, are associated with lower depression scores and reduced levels of SII and UA. These factors collectively contribute to a lower risk of infertility in women. Targeted interventions aimed at enhancing cardiovascular health may potentially mitigate infertility risk through these pathways.

## 1 Introduction

Infertility, characterized by the inability to conceive after at least 12 months of unprotected intercourse (1), affects approximately 15% of couples worldwide (2–4). Beyond its central role in reproductive health, infertility profoundly impacts the physical and mental well-being of affected women, leading to distress, depression, and declining birth rates (5–7). The etiology of infertility is multifaceted, encompassing conditions such as premature ovarian insufficiency (4, 8), polycystic ovary syndrome (PCOS) (1), uterine fibroids, endometriosis (9, 10), and endometrial polyps. Recently, lifestyle factors have gained attention for their potential role in impairing fertility through inflammatory responses (11, 12).

Infertility extends beyond reproductive health, significantly affecting the overall physical, emotional, and social well-being of individuals and families. Shared risk factors between infertility and cardiovascular disease (CVD) include tobacco use, diet quality, and obesity. Thus, linking infertility with these factors could provide valuable markers for early cardiovascular health (CVH) screening and preventive efforts. CVD remains the leading cause of mortality in the United States (13). PCOS, a known cause of infertility, is associated with impaired glucose tolerance and CVD (14). Approximately 25% of female infertility cases are attributed to anovulation related to PCOS, with many cases remaining unexplained. Previous evidence has uncovered a notable correlation between infertility and CVH (15). However, the relationship between CVH metrics and infertility lacks solid evidence, warranting further exploration to elucidate the implications of CVD prevention for women grappling with infertility.

In 2022, the American Heart Association (AHA) introduced Life’s Essential 8 (LE8), incorporating sleep health and expanding upon the previous Life’s Sample 7 (LS7) health domains (16). LE8 categorizes individuals into poor, intermediate, and ideal levels based on eight CVD risk factors and behaviors: smoking, physical activity, BMI, total cholesterol, fasting glucose, blood pressure, diet, and sleep. Research by Wang et al. has shown an inverse relationship between CVH and infertility (17). A recent study also indicated an association between a history of infertility and lower overall and biomedical CVH scores (15). However, the relationship between LE8 and infertility remains underexplored.

This study aims to investigate the relationship between LE8 and infertility among women aged 20-45 in the United States. We hypothesize that inflammation, depression and metabolism mediate this relationship. Through a dissection of the interplay among LE8, inflammation, metabolism, and infertility, our aim is to explicate the roles of inflammation, depression and metabolism in this relationship. Understanding these connections will not only aid in preventing and managing cardiovascular diseases but also inform comprehensive infertility treatment strategies, thereby enhancing overall health outcomes for affected women.

## 2 Methods

### 2.1 Data sources

This study was conducted based on data from the National Health and Nutrition Examination Survey (NHANES) database, a continuous, multistage, cross-sectional health survey program manage by the Centers for Disease Control and Prevention (CDC) to evaluate the health status and nutritional intake of the US population (18). The NHANES program adhered to strict uniform protocols and standards, and researchers received specialized training to ensure the high quality of questionnaire and physical examination data. The NHANES protocol was approved by the National Center for Health Statistics (NCHS) Ethics Review Board, and all participants provided written informed consent (19).

### 2.2 Study design

**Figure 1:**
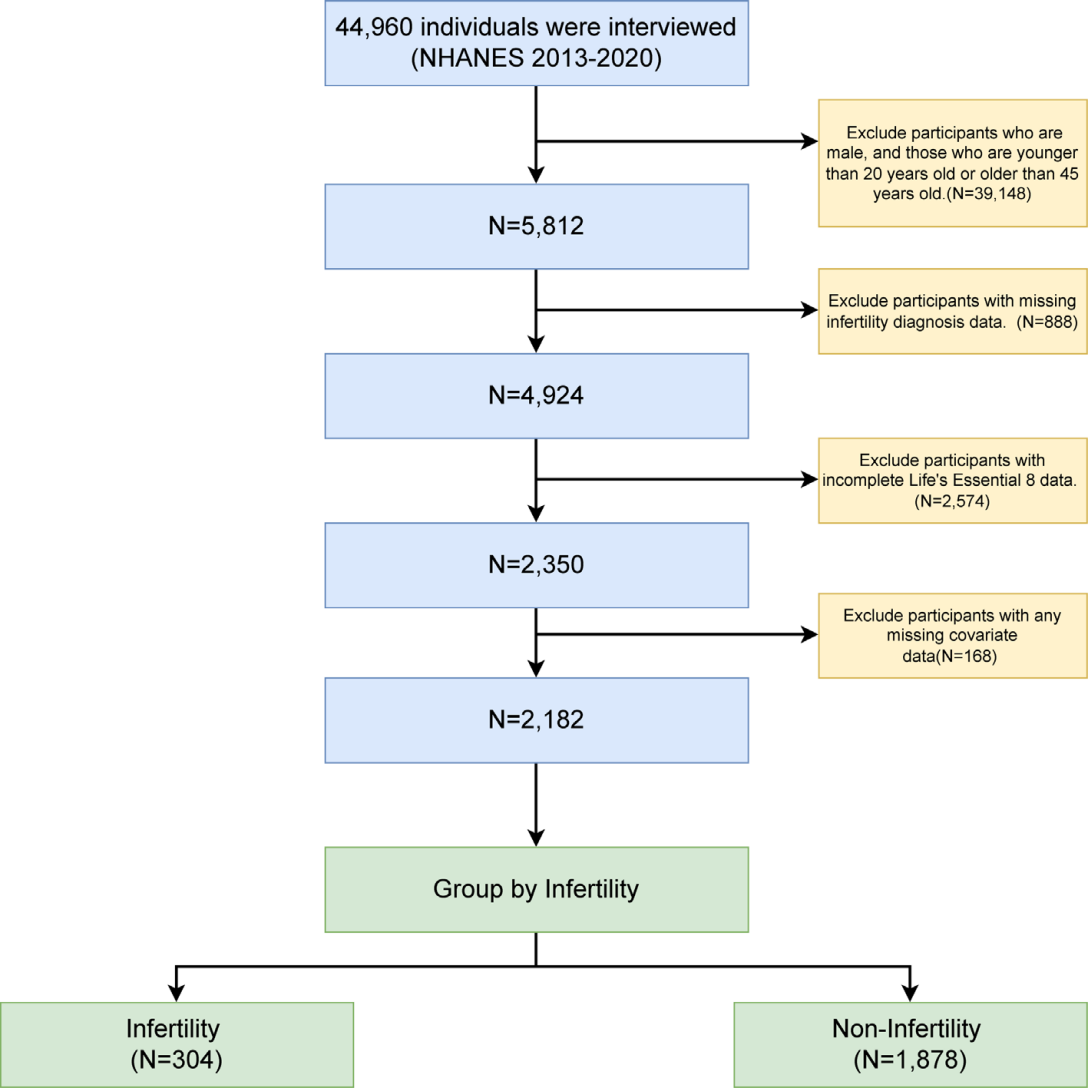
Participant flowchart.

As a cross-sectional study, we strictly followed to the STROBE Statement for cross-sectional studies. Participants from the 2013 to 2020 NHANES were selected for this study. Out of the 44,960 subjects enrolled in NHANES during these years, 5,812 female participants aged 20-45 years were selected based on study requirements. Due to missing infertility diagnosis data, 888 participants were excluded. An additional 2,574 subjects were excluded due to incomplete LE8 score data. Furthermore, 168 participants were excluded for missing covariate information, such as the Patient Health Questionnaire-9 (PHQ-9) scale information, marital status, family economic status, and education. Following these screening criteria, a total of 2,182 participants with complete information were included in the study. Among these participants, 304 had a history of infertility (**Figure 1**).

### 2.3 Data collection and variable definitions

#### 2.3.1 CVH scores

In this study, CVH was assessed according to the LE8 score proposed by the AHA in 2022 (16). The LE8 consists of two subgroups, the health behavior score (HBS) and the health factor score (HFS), which include eight domains: namely, the four HBS domains of diet, physical activity, nicotine exposure, and sleep health, as well as the four HFS domains of body mass index (BMI), lipids, glucose, and blood pressure. Scores for each CVH metric ranged from 0 to 100, and the overall LE8 score was derived by calculating the unweighted mean of the eight CVH metrics (see **Supplementary Table S1** for details of the calculation). Based on the LE8 score, participants were categorized into three cardiovascular fitness level groups, with 80-100 categorized as a high CVH group, 50-79 as a medium CVH group, and 0-49 as a low CVH group. The same cut-off values were used to define HBS and HFS.

#### 2.3.2 PHQ-9 measures and depressive states

The PHQ-9 is a self-administered tool used to assess depressive symptoms and diagnose depression. It consists of nine items, each scored from 0 (not at all) to 3 (nearly every day), resulting in a total score ranging from 0 to 27. Higher scores indicate more severe depressive symptoms. The scoring classification of the PHQ-9 scale for depressive symptoms is as follows:0-4: no depressive state, 5-9: mild depression,10 or more: moderate to severe depressive state (20).

#### 2.3.3 Covariates

Demographic information was collected through household interviews using a standardized questionnaire, which included details on age, race, education, marital status, household income status (PIR, categorized according to poverty income ratios: less than 1.3 for low, 1.3 to less than 3.5 for intermediate, and 3.5 and above for high), and alcohol consumption (g/day). Metabolic indicators included uric acid (UA) levels (mg/dL). The selection of UA as a metabolic indicator is based on its relevance in assessing various health risks. Elevated UA levels, known as hyperuricemia, have been linked to several metabolic disorders and health conditions. These include cardiovascular diseases such as hypertension, coronary artery disease, and stroke, as well as metabolic syndrome components like obesity, insulin resistance, hyperglycemia, hypertension, and dyslipidemia. Moreover, hyperuricemia is associated with an increased risk of chronic kidney disease and can exacerbate complications in patients with diabetes. By measuring UA levels, we aim to comprehensively evaluate participants’ metabolic health status and identify potential health risks (21). Inflammation indicators were selected from the Systemic Immune Inflammation Index (SII), which was calculated from platelet count x neutrophil count/lymphocyte count and expressed as 10^9^ cells per microliter (22).

#### 2.3.4 Definition of outcomes

The diagnosis of infertility history typically hinges on responses to specific inquiries, focusing on two primary aspects: 1. Duration of attempts to conceive: Women are queried about whether they have endeavored unsuccessfully to conceive for a minimum of one year. 2. Medical consultation: Participants are also asked if they have sought medical advice due to difficulties in conceiving. In line with the NHANES questionnaire design, women are classified as having a history of infertility if they respond affirmatively to either of these questions.

### 2.4 Statistical analysis

Statistical analysis was conducted using R software version 4.30. Two-sided tests were employed for statistical inference, with significance set at P ≤ 0.05. Clinical baseline data were summarized using frequencies (weighted percentages) for categorical variables, with group differences assessed using chi-square tests. Continuous variables were presented as mean ± standard error. For normally distributed data, one-way ANOVA was applied, while the Wilcoxon test was used for non-normally distributed data.

Weighted multifactorial logistic regression models were constructed to investigate the association between CVH, HBS, HFS, depressive state, and infertility. To explore potential nonlinear relationships, weighted restricted cubic spline (RCS) regression models were employed. Likelihood ratio test was used to test nonlinearity. The number of nodes was determined based on the lowest value of the Akaike information criterion (AIC).Subgroup analyses were conducted based on race, education level, age, household income, and marital status, with interactions assessed via likelihood ratio tests.

Mediation analyses were performed to evaluate the mediating effect of LE8 scores on infertility, considering depression (PHQ-9), oxidative stress (UA), and inflammation (SII) variables. We used a similar approach to assess the relationships between LE8 and PHQ-9, UA, and SII, as well as the relationships among PHQ-9, UA, SII, and infertility. The R packages ‘mediator’ and ‘lavaan’ were employed to estimate the independent and joint mediating effects of SII, UA, and PHQ-9, respectively. A total of 5,000 replicate simulations were conducted to obtain more accurate estimates of the mediating effects and their confidence intervals. The results included the effect (β), indirect effect (βindirect, β-IE), direct effect (βdirect, β-DE), total effect (Total β), and p-values. Mediation effects were analyzed based on LE8 scores (per 10-point increase).

Two sensitivity analyses were conducted to verify the robustness of the findings. Sensitivity analysis 1 reanalyzed the data using unweighted methods to ensure that results were not dependent on the weighting approach. Sensitivity analysis 2 made further adjustments for variables associated with CVH and infertility, including total bilirubin, creatinine, CVD, albumin, SII, and UA. These additional corrections in sensitivity analysis 2 aimed to account for potential confounding factors.

## 3 Results

### 3.1 Baseline characteristics

The study encompassed 2,182 participants, including 304 individuals in the infertile group. The mean age of participants in the non-infertile group was 32.17 years, significantly lower than the infertile group (35.23 years, p < 0.001). Marital status analysis revealed a higher proportion of individuals in the infertile group living as part of a couple (Coupled) compared to the non-infertile group (p < 0.001). Additionally, infertility prevalence was notably higher among participants in the middle- and high-income brackets (p = 0.008). Regarding physical health indicators, the infertile group exhibited significantly lower scores in HFS, BMI, blood lipids, blood pressure, blood glucose, and sleep health compared to the non-infertile group (p < 0.05). The infertile group also had a higher proportion of low scores in CVH and HFS, along with significantly higher levels of UA and depression scores (PHQ-9). Notably, severe depression was more prevalent in the infertile group compared to the non-infertile group (18.37% vs. 9.8%) (**Table 1**).

**Table 1:** Baseline characteristics according to infertility.

Variable	Total	Non-infertility	Infertility	*P* value
Age, year	32.63 ± 0.25	32.17 ± 0.24	35.23 ± 0.59	< 0.001
Education, n (%)				0.659
Less than high school	91 (2.63)	81 (2.77)	10 (1.85)	
High school	630 (26.56)	542 (26.59)	88 (26.39)	
Some college or above	1461 (70.80)	1255 (70.63)	206 (71.76)	
Ethnicity, n (%)				0.278
White	771 (57.02)	643 (55.97)	128 (62.92)	
Black	463 (12.98)	400 (13.08)	63 (12.42)	
Mexican	371 (12.12)	323 (12.36)	48 (10.82)	
Hispanic	211 (6.92)	191 (7.29)	20 (4.81)	
Other	366 (10.96)	321 (11.30)	45 (9.04)	
Marital Status, n (%)				< 0.001
Coupled	1276 (60.39)	1056 (57.88)	220 (74.54)	
Single or separated	906 (39.61)	822 (42.12)	84 (25.46)	
Family Income, n (%)				0.008
Low	778 (28.86)	680 (30.03)	98 (22.32)	
Intermediate	813 (36.55)	697 (34.92)	116 (45.70)	
High	591 (34.59)	501 (35.05)	90 (31.98)	
LE8	74.60 ± 0.57	75.34 ± 0.58	70.44 ± 1.34	0.001
HBS	69.07 ± 0.70	69.57 ± 0.75	66.25 ± 1.57	0.058
HFS	80.14 ± 0.63	81.12 ± 0.67	74.63 ± 1.56	< 0.001
Body Mass Index	59.45 ± 1.42	61.34 ± 1.39	48.83 ± 3.57	0.001
Blood Lipids	79.83 ± 0.81	80.55 ± 0.86	75.75 ± 2.05	0.033
Blood Pressure	87.50 ± 0.67	88.15 ± 0.71	83.82 ± 1.92	0.042
Blood Glucose	93.78 ± 0.47	94.43 ± 0.51	90.12 ± 1.33	0.005
Sleep Health	84.51 ± 0.68	85.12 ± 0.70	81.09 ± 1.81	0.038
Nicotine Exposure	75.36 ± 1.22	76.19 ± 1.27	70.67 ± 3.06	0.095
Diet	38.56 ± 1.27	38.97 ± 1.35	36.24 ± 2.42	0.288
Physical Activity	77.84 ± 1.22	77.99 ± 1.23	77.00 ± 3.33	0.772
CVH, n (%)				< 0.001
Low (LE8<50)	141 (5.90)	108 (4.93)	33 (11.36)	
Moderate [50-79)	1245 (53.63)	1055 (52.57)	190 (59.64)	
High (≥80)	796 (40.46)	715 (42.50)	81 (29.01)	
HBS, n (%)				0.083
Low (HBS<50)	381 (15.63)	314 (14.85)	67 (20.06)	
Moderate [50-79)	1101 (49.12)	946 (48.72)	155 (51.41)	
High (≥80)	700 (35.24)	618 (36.44)	82 (28.53)	
HFS, n (%)				< 0.001
Low (HFS<50)	142 (5.67)	109 (4.88)	33 (10.12)	
Moderate [50-79)	863 (37.53)	714 (35.79)	149 (47.34)	
High (≥80)	1177 (56.80)	1055 (59.33)	122 (42.53)	
SII	540.72 ± 8.09	541.93± 9.30	533.90 ± 16.43	0.682
UA, mg/dl	4.57 ± 0.03	4.54 ± 0.04	4.78 ± 0.09	0.017
PHQ-9	3.59 ± 0.13	3.39 ± 0.13	4.76 ± 0.45	0.006
Depression Status, n (%)				0.004
Non-Depression	1568 (72.58)	1372 (74.20)	196 (63.47)	
Mild-Depression	386 (16.33)	324 (16.00)	62 (18.17)	
Depression	228 (11.09)	182 (9.80)	46 (18.37)	

CVH, Cardiovascular health; LE8, Life’s essential 8; HBS, Health behaviors; HFS, Health factors; SII, Systemic immune-inflammation index; UA, Uric acid.

### 3.2 Multifactorial logistic regression analysis

The results of multifactorial logistic regression analysis revealed a significant negative association between the HFS within LE8 and infertility (p=0.004), both overall and in the BMI score(p=0.002) and blood glucose score(p=0.030) subgroups. The OR and 95% CI for each 10-point increase in HFS was 0.855 (0.767, 0.954). This indicates that higher cardiovascular fitness, as reflected in higher LE8 scores and increased levels of healthy physical activity, may mitigate the risk of infertility. Conversely, the association between the HBS score and infertility did not reach statistical significance (p=0.077). Furthermore, scores for sleep health, BMI, and blood glucose were significant negative correlations with infertility. In contrast, there was a notable positive correlation observed between depressive symptoms, as measured by PHQ-9 scale scores, and infertility (p<0.001), with an OR and 95% CI of 1.073 (1.031,1.116) for each point increase in PHQ-9, indicating that individuals reporting higher levels of depression symptoms are more prone to infertility (**Table 2**, **Supplementary Table S2**).

**Table 2:** Association of CVH and depression with infertility.

Variable	Model 1		Model 2		Model 3	
	*OR* (95% CI)	*P* value	*OR* (95% CI)	*P* value	*OR* (95% CI)	*P* value
Cardiovascular Health						
Low (LE8<50)	1.000 (1.000,1.000)		1.000 (1.000,1.000)		1.000 (1.000,1.000)	
Moderate [50-79)	0.493 (0.311,0.782)	0.003	0.577 (0.358,0.928)	0.025	0.575 (0.349, 0.947)	0.031
High (≥80)	0.297 (0.167,0.528)	<0.001	0.366 (0.205,0.651)	0.001	0.361 (0.198, 0.660)	0.002
LE8 (per 10)	0.791 (0.697,0.899)	<0.001	0.821 (0.725,0.929)	0.003	0.816 (0.712, 0.935)	0.005
*p* for trend		<0.001		0.002		0.003
Health Behaviors						
Low (HBS<50)	1.000 (1.000,1.000)		1.000 (1.000,1.000)		1.000 (1.000,1.000)	
Moderate [50-79)	0.781 (0.517,1.179)	0.232	0.767 (0.508,1.159)	0.201	0.817 (0.529, 1.260)	0.348
High (≥80)	0.579 (0.328,1.024)	0.060	0.565 (0.324,0.987)	0.045	0.603 (0.332, 1.093)	0.093
HBS (per 10)	0.916 (0.839,1.001)	0.052	0.916 (0.841,0.998)	0.045	0.926 (0.842, 1.018)	0.107
*p* for trend		0.054		0.041		0.077
Health Factors						
Low (HFS<50)	1.000 (1.000,1.000)		1.000 (1.000,1.000)		1.000 (1.000,1.000)	
Moderate [50-79)	0.638 (0.404,1.007)	0.053	0.752 (0.459,1.230)	0.248	0.722 (0.440, 1.186)	0.190
High (≥80)	0.346 (0.196,0.611)	<0.001	0.441 (0.239,0.814)	0.010	0.431 (0.232, 0.801)	0.009
HFS (per 10)	0.819 (0.744,0.902)	<0.001	0.854 (0.769,0.950)	0.005	0.855 (0.767, 0.954)	0.007
*p* for trend		<0.001		0.004		0.004
Depression						
Non-Depression	1.000 (1.000,1.000)		1.000 (1.000,1.000)		1.000 (1.000,1.000)	
Mild-Depression	1.327 (0.830,2.121)	0.230	1.457 (0.910,2.334)	0.114	1.533 (0.934,2.516)	0.088
Depression	2.191 (1.368,3.509)	0.002	2.301 (1.464,3.617)	<0.001	2.549 (1.599,4.065)	<0.001
PHQ-9 (per 1)	1.063 (1.023,1.104)	0.003	1.067 (1.027,1.108)	0.001	1.073 (1.031,1.116)	<0.001
*p* for trend		0.004		0.001		<0.001

Model 1: adjusts for none.

Model 2: adjusts for age and ethnicity.

Model 3: adjusts for age, ethnicity, income, education and marital status. In the model3 that associates cardiovascular health, health behaviors and health factors with infertility, we also adjusted for the PHQ-9. In the association between depression and infertility, we further adjusted for cardiovascular health.

LE8, Life’s essential 8; HBS, Health behaviors; HFS, Health factors; OR, odds ratio; CI, confidence interval.

### 3.3 Dose response relationships

**Figure 2:**
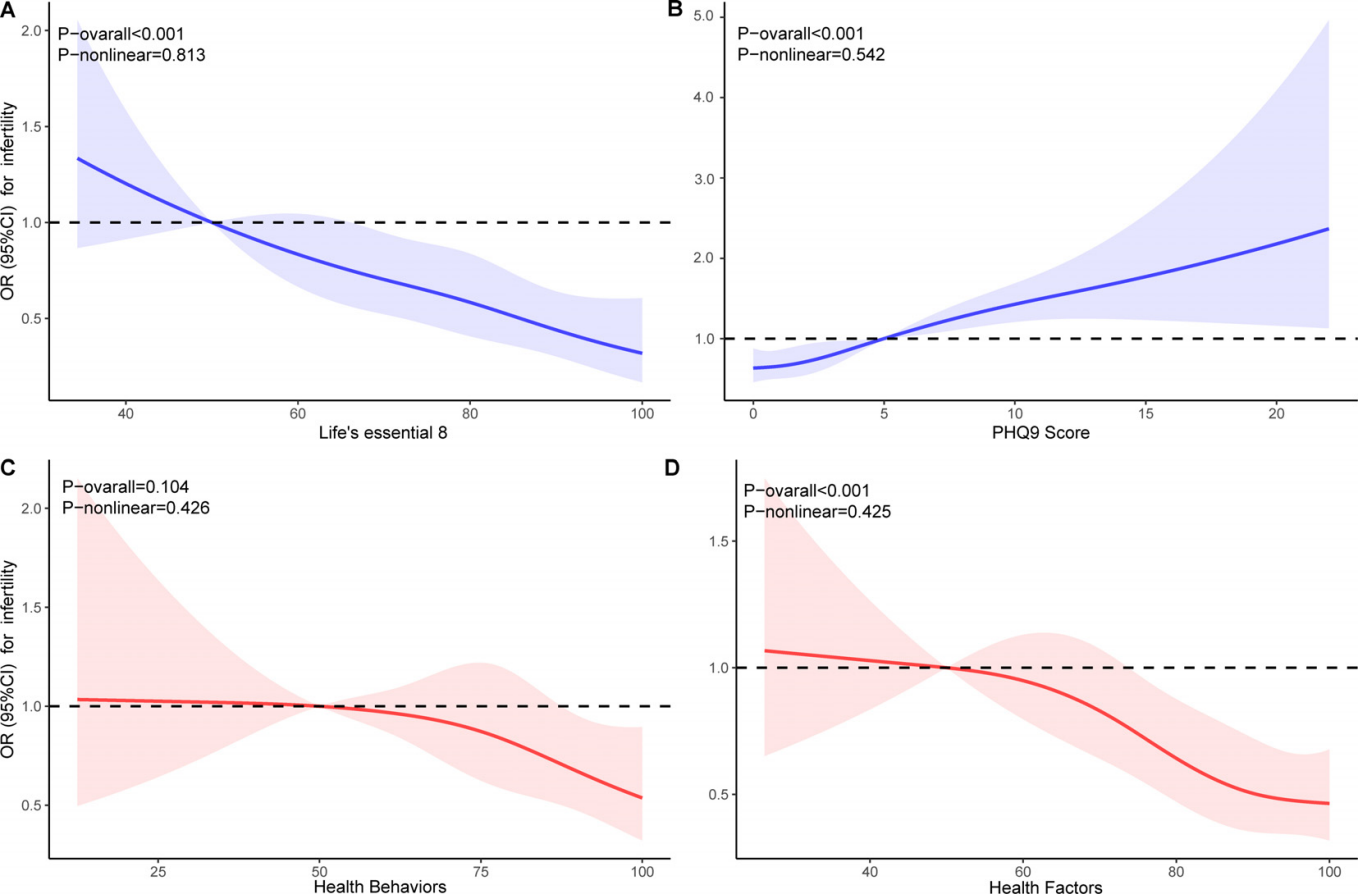
Dose-response relationships for Life’s Essential 8 **(A)**, PHQ-9 **(B)**, Health Behaviors **(C)**, and Health Factors **(D)** with infertility. OR, odds ratio; CI, confidence interval.

RCS analysis indicated that all non-linear associations were found to be statistically non-significant (P-nonlinear > 0.05). LE8 and HFS demonstrated a linear negative correlation (P-overall < 0.05), while PHQ-9 scores showed a linear positive correlation. There was no significant correlation observed with HBS (P-overall > 0.05) (**Figure 2**).

### 3.4 Subgroup analysis

**Figure 3:**
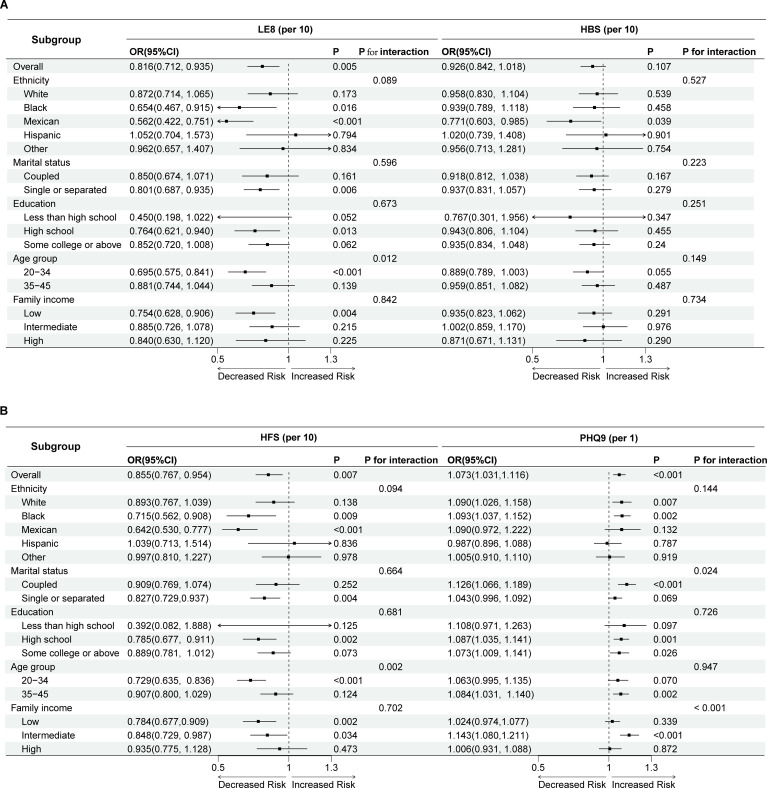
Forest plot of subgroup analysis and interaction tests in Life’s Essential 8, Health Behaviors **(A)**, Health Factors and PHQ-9 **(B)** for the risk of infertility. LE8, Life’s Essential 8; HBS, Health Behaviors; HFS, Health Factors; OR, odds ratio; CI, confidence interval.

The association between LE8 and its subgroup HFS with infertility varies depending on age, with a significant association observed in the 20-34 age group. Additionally, the association between PHQ-9 scores and infertility varies across different stratifications by marital status and income levels. Specifically, a significant association between depression scores and the risk of infertility is found among coupled individuals and those with intermediate income levels, while other groups do not show significant associations (**Figure 3**).

### 3.5 Mediation analysis

**Figure 4:**
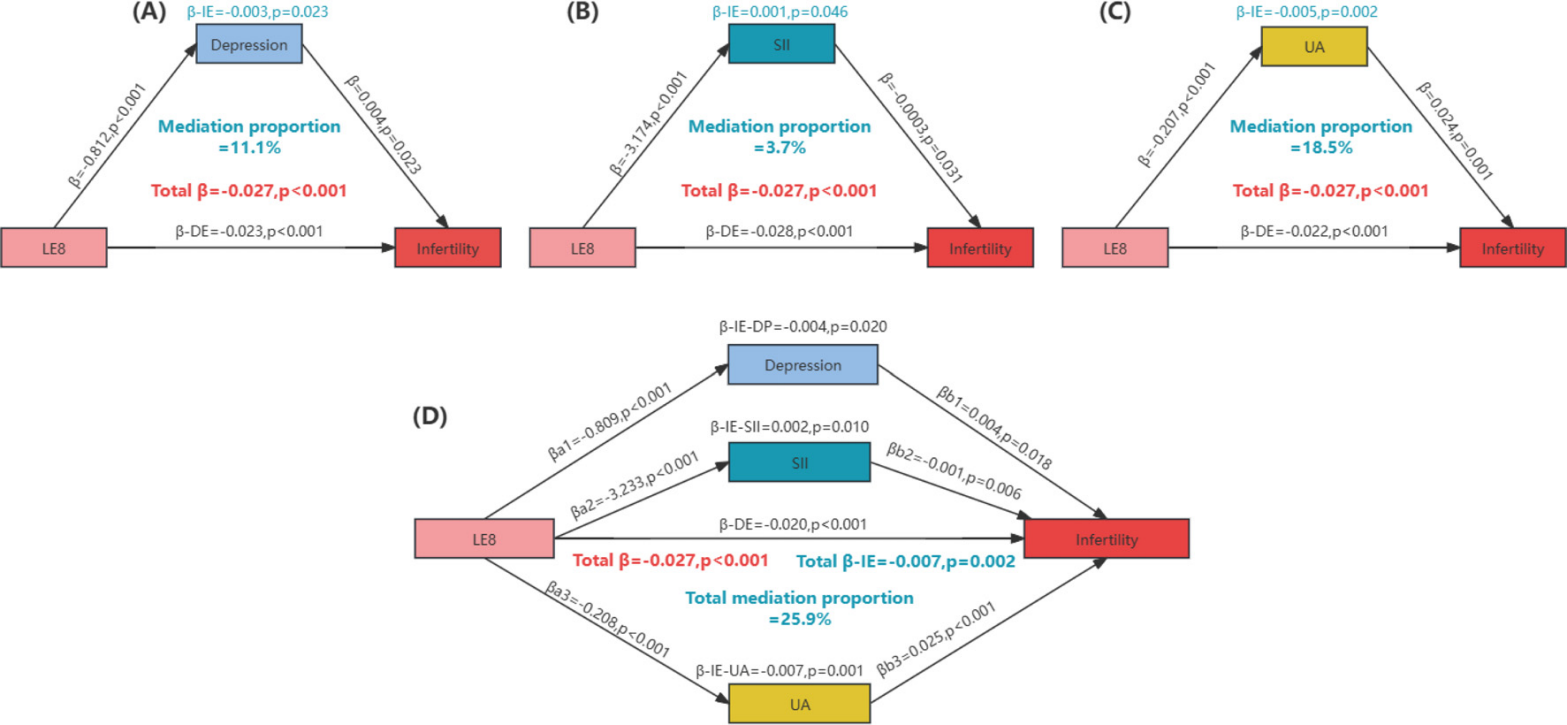
Quantitative assessment of depression, oxidative stress, and inflammation as mediators in the association between life’s Essential 8 and infertility. **(A)** The mediating effect of depression; **(B)** The mediating effect of SII; **(C)** The mediating effect of UA; **(D)** Joint mediating effects of depression, SII and UA. Mediation proportion = β-IE/Total β. IE, indirect effect; DE, direct effect; LE8, Life’s Essential 8; SII, systemic immune-inflammation index; UA, uric acid.

LE8 (per 10-point increase) showed a negative correlation with depression score, SII (1/10 SII), and UA (β-depression=-0.812, p<0.001; β-SII=-3.174, p<0.001; β-UA=-0.207, p<0.001). Additionally, depression score and UA were positively correlated with infertility, while SII was negatively correlated with infertility (β-depression=0.004, p=0.023; β-SII=0.0003, p=0.031; β-UA=0.024, p=0.001). Mediation analyses indicated that depression score, SII, and UA collectively mediated 25.9% of the relationship between LE8 and infertility, with individual mediations of 11.1%, 3.7%, and 18.5%, respectively (p<0.001) (**Figure 4**).

### 3.6 Sensitivity analysis

Sensitivity analysis confirmed the robustness of our results. Both the primary analysis and sensitivity analysis 2 utilized weighted data, while sensitivity analysis 1 employed unweighted data. Adjustments in the primary analysis and sensitivity analysis 1 were made for age, ethnicity, income, education, and marital status, with further adjustments for PHQ-9 scores in cardiovascular health associations and for cardiovascular health metrics in depression associations. Sensitivity analysis 2 included additional adjustments for total bilirubin, creatinine, albumin, uric acid, systemic immune-inflammation index, and history of cardiovascular disease. The consistent findings across these analyses affirm the reliability and stability of the observed associations (**Table 3**).

**Table 3:** Sensitivity analysis of the association of the CVH and depression with infertility.

Variable	Primary Analysis		Sensitivity Analysis 1		Sensitivity Analysis 2	
	*OR* (95% CI)	*P* value	*OR* (95% CI)	*P* value	*OR* (95% CI)	*P* value
Cardiovascular Health						
Low (LE8<50)	1.000 (1.000,1.000)		1.000 (1.000,1.000)		1.000 (1.000,1.000)	
Moderate[50-79)	0.575 (0.349, 0.947)	0.031	0.666 (0.431,1.050)	0.073	0.640 (0.380, 1.077)	0.090
High (≥80)	0.361 (0.198, 0.660)	0.002	0.418 (0.256,0.692)	<0.001	0.435 (0.219, 0.864)	0.020
LE8 (per 10)	0.816 (0.712, 0.935)	0.005	0.814 (0.741,0.894)	<0.001	0.853 (0.743, 0.978)	0.025
*p* for trend		0.003		<0.001		0.027
Depression						
Non-Depression	1.000 (1.000,1.000)		1.000 (1.000,1.000)		1.000 (1.000,1.000)	
Mild-Depression	1.533 (0.934,2.516)	0.088	1.423 (1.027,1.951)	0.031	1.494 (0.906,2.465)	0.111
Depression	2.549 (1.599,4.065)	<0.001	1.879 (1.280,2.719)	0.001	2.662 (1.622,4.368)	<0.001
PHQ-9 (per 1)	1.073 (1.031,1.116)	<0.001	1.051 (1.024,1.079)	<0.001	1.075 (1.030,1.121)	0.002
*p* for trend		<0.001		<0.001		<0.001

The primary analysis and sensitivity analysis 2 used weighted data, while sensitivity analysis 1 used unweighted data.

Primary analysis and sensitivity analysis 1: adjusts for age, ethnicity, income, education and marital status. In the association with cardiovascular health, further adjustments were made for the PHQ-9. In the association with Depression, further adjustments were made for the cardiovascular health.

Sensitivity analysis 2: based on the primary analysis, further adjustments were made for total bilirubin, creatinine, albumin, uric acid, systemic immune-inflammation index, uric acid and cardiovascular disease history.

LE8, Life’s essential 8; OR, odds ratio; CI, confidence interval.

## 4 Discussion

Our study uncovered a novel negative correlation between LE8 and infertility, indicating that elevated cardiovascular fitness levels may mitigate the risk of infertility. We further found that HFS, a subgroup of LE8, was inversely associated with infertility. Among the eight subscales of LE8, scores for sleep health, BMI, and blood glucose were significantly negatively correlated with depression scores, whereas depressive state and PHQ-9 scores were positively associated with infertility. Furthermore, SII, UA and depression were identified as mediators in the relationship between LE8 and infertility, highlighting their significant mediating roles.

Previous research has identified a relationship between CVH and infertility, highlighting increased susceptibility to cardiovascular issues among infertile women. A recent prospective cohort study supported this link by demonstrating lower LE8 scores in association with infertility (15). Furthermore, infertility has been linked to heightened risks of various cardiovascular diseases in women, including CVD events, cardiovascular mortality, coronary heart disease, stroke, and heart failure. Despite these observations, the specific impact of LE8 on infertility remains underexplored. Our study contributed by revealing a negative association between LE8 scores and infertility, suggesting that optimal cardiovascular health may lower infertility risk in women. Research evidence has indicated that specific indicators within the LE8 framework are associated with infertility, including BMI (23), smoking (24, 25), physical activity (26, 27), diet quality (28–30), and dietary fiber (31). Our study further validated associations of sleep health score, blood glucose score, and BMI score with infertility. Consistent with a previous study by Wang et al. (17), fertility was positively correlated with LS7 scores, suggesting reduced infertility odds with improved cardiovascular health. Notably, LE8’s impact on sleep was more significant compared to LS7, aligning with findings that shorter sleep duration increases infertility risk (26, 32, 33). Therefore, LE8 presents promising prospects for managing cardiovascular risks and potentially enhancing fertility outcomes among affected individuals.

Our further analysis revealed a higher prevalence of major depression in the infertility group. In-depth examination demonstrated a significant positive correlation between depressive states and PHQ-9 scale scores with infertility. These findings were consistent with studies in Hungary and Pakistan, which reported higher rates of depression and anxiety among infertile women (34, 35). Infertile women typically exhibit more symptoms of depression and anxiety compared to their partners (36). The Iranian study highlighted that infertile women often experience psychological issues, such as sexual dysfunction, due to depression and anxiety (37). Additionally, research indicates that PHQ-9 scores may influence infertility by impacting BMI (38). Infertility presents significant emotional challenges, leading to various psychological issues, including anxiety, depression, eating disorders, and low self-esteem. Interventions like psychological counseling and therapy for infertile couples have demonstrated substantial reductions in these psychological issues and have been shown to enhance conception rates (39). Thus, providing psychological support and treatment for infertile women is crucial.

PCOS is a leading cause of infertility often linked with insulin resistance and cardiometabolic issues like dyslipidemia, hypertension, impaired glucose tolerance, diabetes mellitus, and metabolic syndrome (MetS), increasing cardiovascular disease risk in affected women. Research has shown that obesity significantly raises the risk of infertility. Obese individuals frequently exhibit functional hyperandrogenism and hyperinsulinemia, which, together with insulin resistance, impair fertility (40). Additionally, obesity triggers oxidative stress and ovarian inflammation, stimulating the production of androgens, while enhancing peripheral aromatization of estrogen and reducing the production of sex hormone-binding globulin (SHBG) in the liver. These disruptions lead to follicular atresia and anovulatory cycles (41). Studies have also demonstrated that women of normal or overweight BMI have significantly higher fertility rates compared to obese women, suggesting that obesity may not only impair oocyte quality but also negatively affect uterine receptivity, further compounding fertility challenges (42).

Furthermore, elevated blood glucose scores were found to be inversely associated with infertility risk. Higher levels of blood glucose and hemoglobin A1c have been linked to an increased likelihood of infertility in women. Appropriate glucose levels are essential for the maturation of oocytes and successful embryo development. During the nuclear and cytoplasmic maturation of oocytes, abnormal glucose concentrations can disrupt these processes, leading to poor embryo development after fertilization and reduced fertility (43). Our study identified a negative association between blood glucose scores and infertility risk. Observational studies suggest impaired glucose tolerance links to both cardiovascular risk and fertility problems. Elevated blood glucose levels correlate with pregnancy complications, infertility, and miscarriage risk if poorly managed. Shared pathogenic mechanisms between infertility and cardiovascular disease include activation of the hypothalamic-pituitary-adrenal (HPA) axis and neuroendocrine stress responses. Hyperuricemia indeed represents a hallmark of the metabolic syndrome (44). UA played an 18.5% mediating role between LE8 scores and infertility in our mediation analysis, underscoring metabolism’s critical role linking cardiovascular health and infertility. Infertility thus serves as a potential indicator for cardiometabolic disorders, warranting timely interventions to mitigate their impacts. Women with PCOS may develop adverse cardiovascular traits during reproductive years, affecting both maternal and offspring health outcomes.

Inflammation and immune responses are critical factors in the development of female infertility. Our study investigated the relationship between the SII within LE8 scores and infertility. We found a negative correlation between SII and infertility, consistent with previous research (45). Mediation analysis indicated that SII mediated 3.7% of the association between LE8 scores and infertility. Chronic endometritis, often detected during screening for secondary amenorrhea and infertility (46), may contribute to infertility by disrupting menstrual cycles and impairing implantation. Anti-inflammatory dietary interventions, such as the Mediterranean diet rich in monounsaturated fatty acids, n-3 polyunsaturated fatty acids, and flavonoids, and low in red and processed meat, have shown promise in improving male fertility and sperm quality (12). Implementing an anti-inflammatory diet as an adjunctive treatment for fertility may enhance reproductive outcomes and potentially reduce the need for more intensive medical interventions.

Importantly, the findings suggested that targeted interventions aimed at improving CVH, as indicated by LE8 scores, could have offered potential pathways to mitigate infertility risk. For instance, strategies focused on improving sleep quality, maintaining a healthy BMI, and controlling blood glucose levels might have reduced depression and inflammation, which were significant mediators in the LE8-infertility association. Future research should investigate how specific interventions targeting these health domains could directly influence reproductive outcomes. Moreover, our findings paved the way for interdisciplinary studies to explore how improving cardiovascular health metrics could potentially benefit both cardiovascular and reproductive health. Developing comprehensive health programs that integrated cardiovascular health with fertility treatment might offer new avenues for reducing infertility risk and improving overall women’s health outcomes.

We utilized the NHANES database to conduct our study, leveraging its large sample size to enhance statistical power and result generalizability. Employing multifactorial regression and restricted cubic spline modeling, we explored the association between LE8 scores and infertility, conducting subgroup and mediation analyses to uncover physiological and metabolic mechanisms. Despite the robustness of NHANES data, limitations include potential biases from missing or incomplete data and reliance on self-reported information and physiological measurements, which could introduce errors and biases affecting study accuracy. Given the constraints of observational studies, we couldn’t establish causality between LE8 scores and infertility, necessitating future long-term follow-up or intervention studies. These studies should investigate whether improving cardiovascular health via LE8 scores can mitigate infertility risks. It’s recommended that future interventions target cardiovascular health improvements and assess their long-term impacts on physical and psychological health in infertile patients. Collaborative interdisciplinary efforts are crucial to advancing understanding of cardiovascular disease and infertility mechanisms, supporting the development of effective clinical strategies. In conclusion, while our NHANES-based study provides insights into LE8 scores and infertility, further research is essential to address limitations and translate findings into clinical applications for enhancing women’s health and reproductive management.

## 5 Conclusion

Our study utilizing data from the NHANES database highlights that higher LE8 scores were significantly associated with lower depression scores and reduced levels of SII and UA. These factors collectively contributed to a decreased risk of infertility in women. Notably, improvements in specific LE8 health factors—such as sleep health, BMI and blood glucose management—were shown to have potential for lowering infertility risk by reducing associated mediators like depression and inflammation. Based on the findings of this study, we recommend the development of health policies focused on weight management and glycemic control to help prevent infertility.

## Data Availability

The study’s original contributions are publicly available. You can access this data at the NHANES website: https://www.cdc.gov/nchs/nhanes.
