# Supplementary material for: Deciphering the association between the Life’s Essential 8 and infertility: insights into depression, inflammation, and metabolic mediation
Source: Front Endocrinol (Lausanne). 2025 Feb 21;16:1451030. doi: 10.3389/fendo.2025.1451030 (PMC11885257; doi:10.3389/fendo.2025.1451030)
Supplement: Supplementary file 1 [file Table1.docx]

**Table S1** Definition and scoring approach for the American Heart Association’s Life’s Essential 8 score.

| **Domain** | **CVH Metric** | **Measurement** | **Quantification and Scoring of CVH Metric** |
| --- | --- | --- | --- |
| Health Behaviors | Diet | Healthy Eating Index-2015 diet score percentile | Quantiles of DASH-style diet adherence  Scoring (Population):  Points Quantile  100 ≥95^th^ percentile (top/ideal diet)  80 75^th^ – 94^th^ percentile  50 50^th^ – 74^th^ percentile  25 25^th^ – 49^th^ percentile  0 1^st^ – 24^th^ percentile (bottom/least ideal quartile) |
|  | Physical activity | Self-reported minutes of moderate or vigorous physical activity per week | Metric: Minutes of moderate (or greater) intensity activity per week  Scoring:  Points Minutes  100 ≥150  90 120 – 149  80 90 – 119  60 60 – 89  40 30 – 59  20 1 – 29  0 0 |
|  | Nicotine exposure | Self-reported use of cigarettes or inhaled nicotine- delivery system | Metric: Combustible tobacco use and/or inhaled NDS use; or secondhand smoke exposure  Scoring:  Points Status  100 Never smoker  75 Former smoker, quit ≥5 yrs  50 Former smoker, quit 1 - <5 yrs  25 Former smoker, quit <1 year, or currently using inhaled NDS  0 Current smoker  Subtract 20 points (unless score is 0) for living with active indoor smoker in home |
|  | Sleep health | Self-reported average hours of sleep per night | Metric: Average hours of sleep per night  Scoring:  Points Level  100 7 – <9  90 9 – <10  70 6 – <7  40 5 – <6 or ≥10  20 4 – <5  0 <4 |
| Health Factors | Body mass index | Body weight (kg) divided by height squared (m^2^) | Metric: Body mass index (kg/m^2^)  Scoring: Points Level 100 <25  70 25.0 – 29.9  30 30.0 – 34.9  15 35.0 – 39.9  0 ≥40.0 |
|  | Blood lipids | Plasma total and HDL-cholesterol with calculation of non-HDL-cholesterol | Metric: Non-HDL-cholesterol (mg/dL)  Scoring:  Points Level  100 <130  60 130 – 159  40 160 – 189  20 190 – 219  0 ≥220  If drug-treated level, subtract 20 points |
|  | Blood glucose | Fasting blood glucose or casual hemoglobin A1c | Metric: Fasting blood glucose (mg/dL) or Hemoglobin A1c (%)  Scoring:  Points Level  100 No history of diabetes and FBG <100 (or HbA1c < 5.7)  60 No diabetes and FBG 100 – 125 (or HbA1c 5.7-6.4) (Pre-diabetes)  40 Diabetes with HbA1c <7.0  30 Diabetes with HbA1c 7.0 – 7.9  20 Diabetes with HbA1c 8.0 – 8.9  10 Diabetes with Hb A1c 9.0 – 9.9  0 Diabetes with HbA1c ≥10.0 |
|  | Blood pressure | Appropriately measured systolic and diastolic blood pressure | Metric: Systolic and diastolic blood pressure (mm Hg)  Scoring:  Points Level  100 <120/<80 (Optimal)  75 120-129/<80 (Elevated)  50 130-139 or 80-89 (Stage I HTN)  25 140-159 or 90-99  0 ≥160 or ≥100  Subtract 20 points if treated level |

Reference: Lloyd-Jones DM, Allen NB, Anderson CAM, et al. Life's Essential 8: Updating and Enhancing the American Heart Association's Construct of Cardiovascular Health: A Presidential Advisory from the American Heart Association. *Circulation*. Aug 2 2022;146(5): e18-e43.

Table S2 Associations of the 8 subscales with infertility.

| **Variable** | **Model 1** | |  | **Model 2** | |  | **Model 3** | |
| --- | --- | --- | --- | --- | --- | --- | --- | --- |
|  | ***OR*(95% CI)** | ***P* value** |  | ***OR*(95% CI)** | ***P* value** |  | ***OR*(95% CI)** | ***P* value** |
| **Diet** | 0.973(0.923,1.025) | 0.293 |  | 0.963(0.913,1.015) | 0.156 |  | 0.962(0.913,1.013) | 0.135 |
| **Physical Activity** | 0.993(0.950,1.039) | 0.769 |  | 1.000(0.956,1.046) | 0.999 |  | 1.003(0.959,1.050) | 0.883 |
| **Nicotine Exposure** | 0.966(0.930,1.004) | 0.080 |  | 0.971(0.932,1.011) | 0.145 |  | 0.959(0.916,1.004) | 0.072 |
| **Sleep Health** | 0.935(0.882,0.992) | 0.026 |  | 0.923(0.870,0.979) | 0.009 |  | 0.924(0.868,0.984) | 0.016 |
| **Body Mass Index** | 0.915(0.868,0.964) | 0.001 |  | 0.920(0.872,0.970) | 0.003 |  | 0.916(0.868,0.967) | 0.002 |
| **Blood Lipids** | 0.937(0.886,0.991) | 0.025 |  | 0.963(0.906,1.023) | 0.214 |  | 0.964(0.907,1.026) | 0.239 |
| **Blood Glucose** | 0.882(0.819,0.951) | 0.002 |  | 0.911(0.840,0.988) | 0.025 |  | 0.912(0.839,0.991) | 0.030 |
| **Blood Pressure** | 0.922(0.858,0.990) | 0.027 |  | 0.963(0.887,1.045) | 0.354 |  | 0.955(0.877,1.040) | 0.279 |

Model 1: adjusts for none.

Model 2: adjusts for age and ethnicity.

Model 3: adjusts for age, ethnicity, PHQ9, income, education and marital status. In the association between depression and infertility, we further adjusted for cardiovascular health.

OR, odds ratio; CI, confidence interval.
